# How do clinicians rate patient’s performance status using the ECOG performance scale? A mixed-methods exploration of variability in decision-making in oncology

**DOI:** 10.3332/ecancer.2019.913

**Published:** 2019-03-28

**Authors:** Soumitra S Datta, Niladri Ghosal, Rhea Daruvala, Santam Chakraborty, Raj Kumar Shrimali, Chantalle van Zanten, Joe Parry, Sanjit Agrawal, Shrikant Atreya, Subir Sinha, Sanjoy Chatterjee, Simon Gollins

**Affiliations:** 1 Department of Palliative Care and Psycho-oncology, Tata Medical Centre, Kolkata 700160, India; 2 UCL EGA Institute for Women’s Health, University College London, London WC1E 6BT, UK; 3 Department of Clinical Oncology, North Wales Cancer Center, Rhyl LL18 5UJ, UK; 4 Department of Radiation Oncology, Tata Medical Centre, Kolkata 700160, India; 5 Department of Economics and Business, University of Groningen, 9712 CP Groningen, Netherlands; 6 Newcastle University Business School, University of Newcastle, Newcastle upon Tyne NE1 7RU, UK; 7 Department of Surgical Oncology, Tata Medical Centre, Kolkata 700160, India; 8 Department of Biostatistics, Tata Medical Centre, Kolkata 700160, India

**Keywords:** decision making, ECOG, oncology, heuristics

## Abstract

**Background:**

Medical decisions made by oncology clinicians have serious implications, even when made collaboratively with the patient. Clinicians often use the Eastern Clinical Oncology Group (ECOG) performance status (PS) scores to help them make treatment-related decisions.

**Methods:**

The current study explores the variability of the ECOG score when applied to 12 predetermined specially designed clinical case vignettes presented to a group of oncology clinicians (*n* = 72). The quantitative analysis included evaluation of variability of ECOG PS scores and exploration of rater and patient-related factors which may influence the final ECOG rating. In-depth interviews were conducted with oncology clinicians to ascertain factors that they felt were important while making treatment-related decisions. Basic and global themes were generated following qualitative data analysis.

**Results:**

Quantitative results showed that there was poor agreement in ECOG rating between raters. Overall concordance with the gold standard rating ranged between 19.4% and 56.9% for the vignettes. Moreover, patients deemed to have socially desirable qualities (*p* < 0.004) were rated to have better PS and women patients (*p* < 0.004) to have worse PS. Clinicians having international work experience had increased concordance with ECOG PS rating. Qualitative results showed that ‘perceived socio-economic background of the patient’, ‘age of the patient’, ‘patient’s and family’s preferences’ and ‘past treatment response’ were the major themes highlighted by respondents that influenced the treatment-related decisions made by clinicians.

**Conclusion:**

There is considerable variability in ECOG PS determined by clinicians. Decision-making in oncology is complex, multifactorial and is influenced by rater and patient-related factors.

## Background

Decision-making in oncology is often difficult and is influenced by patient preferences, availability and affordability of viable treatment options, views of the treating clinicians and also cultural factors [1]. There can be complex social and ethical dilemmas that need to be tackled when making treatment decisions [2]. Choice of treatment algorithms in oncology is based on performance status (PS) as this is the single most important prognostic factor in oncology. Eastern Clinical Oncology Group (ECOG) PS, first published in 1982 [3], is routinely used in daily clinical practice, as well as in clinical trials. In clinical trials, it forms part of the eligibility criteria.

The other popular method for assessing general fitness or PS was described by Karnofsky in 1949 [4]. A few of the common situations where PS is reported are to determine the general fitness and suitability of patients for treatment, whether palliative or radical; choosing treatment based upon effectiveness balanced with potential toxicity as compared to other available treatment modalities (surgery, radiotherapy, chemotherapy on their own or combined). In the context of clinical trials, it is used to ensure a degree of homogeneity with respect to the PS of the study population. Scoring the patient on the ECOG PS enables different specialties in oncology to communicate effectively and objectively at multi-disciplinary team meetings. In present day practice, we are moving towards a multi-disciplinary team guided management system for patients. Treatment may be determined depending on second-hand descriptions of the patients’ PS and hence reproducibility of ECOG PS is essential. Thus, ECOG PS is also meant to enable different professional groups, such as doctors, physiotherapists, dieticians and specialist nurses to offer and provide appropriate expertise to suit individual patient needs. Therefore, it is extremely important that understanding and application of the PS is correct and reproducible.

To make all this a valid assessment of patients, the underlying tenet is that at any given point in time, all clinicians would score the same patient identically using ECOG. The clinician scoring PS using ECOG is expected to have full knowledge of the tool and apply it correctly. The ECOG PS has stood the test of time because of its simplicity and ease of use with a wide spectrum of patients. However, questions may be raised on its reproducibility, as flexibility of the scoring system may lead to inter-observer variability. Subjectivity may be introduced by various factors that relate to the clinician or the patient.

A European study reported interesting inter-observer variability but concluded that agreement with regard to allocation of patients to PS 0–2 versus 3–4 was high [5]. The coincidence degree between physicians reporting the ECOG and Karnofsky PS in 100 consecutive patients was 59% and 70%, respectively [6]. This study also looked at the correlation between physicians’ and patients’ evaluations of PS. The Kendall’s correlation was 0.65 and 0.61 for physician 1 and 0.65 and 0.46 for physician 2 for patients aged <65 year or >65 years, respectively. This shows a trend of more disparity between the physicians and patient’s self-rated evaluation of ECOG PS with increasing age although this probably did not reach statistical significance with the given sample size.

Previous research has indicated that using clinical vignettes was an inexpensive way of measuring quality of health care delivery in an out-patient setting and is a valid and comprehensive method that reflects the process of care provided in actual clinical practice [7]. Clinical vignettes have been widely used around the world in assessing quality of clinical encounters in a variety of sub-specialities of medicine [8, 9].

In the current study, which had two parts, we initially conducted a questionnaire-based survey with clinicians across specialties in oncology who are directly involved in patient care and followed this up with an in-depth qualitative interviewing of cancer clinicians to understand decision-making in oncology. The survey was designed to identify and quantify inter-observer concordance between cancer clinicians while using the ECOG PS (PS) of cancer patients and to explore if there were any systematic factors that influence the rating such as years of experience as a cancer clinician, age of the clinician, age of the patient, type of cancer and overall social desirability of the patient. The study was designed such that it could elicit some factors that may be associated with common sources of inter-observation variation. As we were expecting that we may not be able to capture all the reasons for the variations that we find, following a sequential mixed-methods design, we included a qualitative part of the study using in-depth interviewing of clinicians working in the field of oncology. To our knowledge, this is the first study using a mixed-methods design to explore decision-making with ECOG PS by cancer clinicians.

## Methods

We studied decision-making by oncology clinicians in a developing country setting following a mixed method of enquiry intertwining qualitative and quantitative research methodology. Several hypothetical but commonly encountered scenarios in oncology were presented to clinicians in the form of case vignettes (Appendix II). The study was conducted after obtaining approval from the Institutional Ethics Committee (EC/TMC/48/15). Written informed consent was obtained from all participants before recruitment.

## Setting

Tata Medical Centre, Kolkata, located in eastern India, served as the study setting and is a tertiary care cancer hospital equipped with almost all the clinical sub-specialties of oncology, including surgical oncology, radiotherapy, medical oncology, clinical haematology, nuclear medicine, radio-diagnosis and palliative medicine. The Department of Surgical Oncology at the hospital comprised of specialised units dedicated to breast cancer surgery, head and neck cancer surgery, gastrointestinal and hepatobiliary surgery, plastic surgery, gynaecological oncology and urology-oncology.

The researchers, who undertook quantitative and qualitative interviews, were not employees of the hospital where the study was conducted. This made them particularly suitable as interviewers, as most clinicians could open up easily to them. All clinicians who were interviewed had studied medicine in English and were fluent in English, which was the preferred language for inter-professional communication and documentation of patient records in the hospital where the study was conducted.

## Research team

The core research team consisted of a consultant psycho-oncologist, one psycho-oncology fellow, four consultant clinical oncologists, a consultant surgical oncologist, a consultant radiation oncologist, a consultant palliative medicine physician, an expert in biostatistics and two visiting social science interns with a background in management and health economics. All the senior members of the team were research active clinicians and were involved in making treatment-related decisions for a wide range of cancer patients. Two of the senior members of the research team had independently led and published qualitative and mixed-methods research, while all other members had undergone training in research methods. The data collection and interviews were conducted by the visiting interns under the supervision of the principal investigator and psycho-oncology fellow. The interviewers had limited if any interaction with the study participants prior to the commencement of the project. This ensured that participants were not inhibited in expressing their views about decision-making and related areas. The senior researchers trained all the interviewers in conducting in-depth interviews prior to data collection.

### Creating the clinical vignettes

The vignettes were created by the study team that comprised of three consultant clinical oncologists and two psycho-oncologists. The vignettes were first decided based on the common clinical scenarios that oncology clinicians encounter in day-to-day medical practice. The steps of creating the clinical vignettes were adapted from those suggested by Kathiresan and Patro [10] and included the following steps: (a) discussion about the common case scenarios that are encountered by oncology clinicians (b) Coming up with the initial clinical ‘story’ (c) Further refinement and discussion if adequate and sufficient information is present or not for rating the vignette. The authors decided on the ‘right’ amount of information that is usually available in a real-life clinical setting and not too elaborate so as to make the scenario unrealistic.

### Arriving at a gold standard rating for the vignettes

As ECOG is used primarily by oncologists, the gold standard rating of the ECOG PS was decided by the three oncologists who were also involved in designing the vignettes. Using an expert consensus on deciding the gold standard response to a question posed through a clinical case vignette has been used in other previous research projects [7]. A consensus rating for each of the 12 vignettes was obtained and this was coordinated by another researcher. Each of the oncologists was requested to rate the clinical case vignette, initially without knowing the rating of the other. The discordance in the ratings in a minority of vignettes was resolved in the second round of feedback and discussions. The oncologists who were part of the study team agreed on a consensus rating at the end of the exercise.

### Attributing and assessing social desirability of the vignettes

Social desirability of people has been researched in various contexts. Characteristics that have been attributed to people with socially desirable qualities include willingness to help others, keeping the interest of others in mind while embarking on any task, being a good listener, willingness to admit a mistake and being embedded in the larger social matrix [11]. Each vignette was rated to be on a person deemed to be ‘socially desirable’ or ‘socially neutral’ by the entire study team, including the psycho-oncologists and oncology clinicians, e.g. being an elderly man who is active and looks after his grandchildren, a young man who is a footballer, a lady who was previously fit and recently struggling with her household chores, a middle-aged lady with breast cancer who is feeling better in between chemotherapy sessions were all deemed to be socially desirable. Examples of ‘socially neutral’ vignettes were ‘a 67-year-old man with lung cancer who is breathless’, ‘a 77-year-old retired teacher who spends his time in reading and takes a driver to drive him to the market’ etc. No one was deemed to be socially undesirable but only rated as socially desirable or neutral. This is in keeping with the professional non-judgmental attitude that clinicians ought to take in ideal circumstances. A consensus rating on social desirability was made by the two psycho-oncologists and all the oncologists who were part of the study team. Each of them rated the vignettes as socially desirable or neutral and any discordance in the ratings was resolved in the second round of feedback and discussions.

### Procedures

A comprehensive list of all doctors and nurses working in the clinical departments of the hospital who came into direct patient contact was obtained from the department of human resources. Potential participants were approached individually by the researchers. Following a sequential design, a proportion of the respondents who participated in the quantitative part of the study were purposively chosen for qualitative in-depth interviews.

### ECOG rating from clinicians

The clinicians working in the hospital were approached for being included in the study. They were interviewed in private, in their own offices at a time suitable to them. Clinicians were requested to rate the vignettes on the ECOG PS. In order to avoid the rare problem of being unsure of any of the items of ECOG rating scale during the data collection, we had the ECOG rating scale (vide Appendix I) with scoring criteria in front of the participant while he/she rated each vignette. This ensured that the participants could fully concentrate on the rating of the vignette rather than feel they were being evaluated on their knowledge of the ECOG rating scale as this was not the purpose of the study.

### Qualitative in-depth interviewing

On completion of the ECOG rating, some clinicians were interviewed in-depth. They were interviewed in a relaxed environment. Clinicians who specialised in various clinical specialties of oncology were invited to participate. Participants were interviewed in their own offices as per their convenience. Respondents were asked about their decision-making processes and how they usually processed the information regarding a patient’s health, disease and daily life in order to come up with the ECOG PS rating in a busy clinic. The qualitative part of the study adhered to the Consolidated Criteria for Reporting Qualitative Research (COREQ) guidelines for research [12]. All qualitative interviews were recorded and transcribed verbatim. The interviewers were trained in the techniques of qualitative in-depth interviewing by a senior researcher of the team.

## Eligibility criteria

All doctors and nurses who had been employed in the hospital and were in direct patient contact were eligible to participate in the study. All clinicians in the study hospital who offered treatment to patients were familiar with ECOG rating and used it routinely. We specifically included only those clinicians who were involved in face-to-face decision-making for patients with cancer. Non-clinical specialties such as laboratory-based disciplines like biochemistry, histopathology and others that were not involved directly in making decisions for treatment based on ECOG rating and were therefore not included in the study.

## Data collection instruments

### Quantitative data

A predesigned set of 12 clinical case vignettes of variable complexity was designed by senior oncologists who were part of the study team. Participants were required to rate each of the cases on the ECOG scale. The case scenarios were presented with the standard ECOG scale which is a categorical scale (0, 1, 2, 3, and so on). Usually, it is easy to identify a PS ECOG grade ‘0’, as well as a grade ‘4’. The difficulty appears to be differentiating between grades 1, 2 or 3. The sample case vignettes were designed to study results of ECOG assessment in patients in all PS grades but more specifically within the grades 1, 2 and 3. Socio-demographic variables were collected using a socio-demographic sheet given to all participants.

### Qualitative data

Interviewers started with a predetermined set of interview items related to decision-making in oncology and the use of the ECOG scale. The interviewer also probed clinicians about decision-making, the algorithms they used and the thought process they engage in while planning and discussing oncology treatments among peers, colleagues and with patients. Qualitative data analysis and data collection went on concurrently in order to incorporate newly emerging themes from earlier interviews until data saturation was achieved.

## Statistical analysis

### Quantitative data analysis

Quantitative analysis comprised of calculation of agreement indices for the entire population and for subsets of the population. Graphical exploration of the agreement was performed using heatmaps. Prespecified subsets for which agreement was calculated included age group, gender, profession type and years of experience in the field of oncology. Additionally, the questionnaire also included a question about the participant’s opinion on the ECOG rating being perceived as a subjective or objective measure. We calculated agreement metrics for this question as well. As multiple raters were involved, a model-based kappa was calculated using the methodology proposed by Nelson *et al* [13]. A kappa of 1 is interpreted as perfect agreement and 0 being absolute disagreement between raters. In order to circumvent the problems of Cohen’s kappa value being influenced by small number values that are different from the other values specially with symmetrical data (kappa paradox), Gwet [14] proposed first-order agreement coefficient or the AC1 statistic, which adjusts for the fact that multiple raters may agree on a rating by chance and addresses the kappa paradox. To describe in brief, this method uses a generalised linear mixed model to calculate the observed agreement between raters while minimising the impact of a chance agreement. Unlike Cohen’s kappa and other variations, this method is not affected by prevalence rates. Furthermore, measure of association was also calculated using the same model-based approach. The function for calculation of the model based kappa was provided by Nelson *et al* [13, 15].

In order to investigate the effect of patient-related factors on the ECOG PS rating, a cumulative link mixed model (CLMM) was constructed for the data. All the patients described in the vignettes were *a priori* assigned to having socially desirable characteristics (vignettes 1, 2, 6 & 7) or neutral characteristics (rest of the vignettes). To give an example—‘a grandmother who played with her grandchildren’ was deemed to have socially desirable characteristics by the researchers and ‘a politician who has lost his voice’ was deemed to be neutral or not specifically having any socially desirable quality in the description in the vignette. Three characteristics of the patient, namely, age of the patient in the vignette, gender of the patient and the perceived social desirability of the patient were fitted into the CLMM. In the same way, CLMM allowed us to explore the effect of individual raters on the PS rating and to explore the way groups of raters tend to rate the PS. Dot plot of the effect of the rater characteristics on the PS rating was also calculated using CLMM faceted as per rater categories. Dots are coloured as per the job role, for example, consultant, trainee doctor or nursing staff. Maximum likelihood estimates of the coefficients were calculated using adaptive Gauss-Hermite quadrature method using 10 quadrature nodes. The exponents of the coefficients allowed us to get the odds ratio of rating being rated for the aforementioned vignettes.

The second part of the analysis focused on the agreement with the gold standard ratings. In this analysis, the proportion of raters who gave the correct rating was the endpoint. Given that ECOG PS is an example of ordinal rating scale, no efforts were made to calculate the distance of disagreement. We also explored if the proportion of agreement with the golden rating varied as per the rater characteristics using the variables mentioned above.

### Qualitative data analysis

Qualitative data analysis went side by side with data collection as is recommended for qualitative research. Qualitative data analysis followed the principles of thematic analysis as suggested by Braun and Clarke [16]. The various steps we used included becoming familiar with the data, generating initial codes, searching for themes or charting data, review of themes and synthesis, generating basic and organising themes and finally, arriving at global themes which defines the data [17]. According to experts, thematic analysis is a method of identifying, analysing and reporting patterns or themes within qualitative data and is able to provide a rich description of the data [16]. They also suggest that one of the advantages of this approach is its flexibility and that thematic analysis should be seen as a foundational method for qualitative analysis [17].

## Results

### Quantitative results

#### Respondents

The questionnaire was administered to a total of 72 participants. The mean age of the respondents was 33.4 years (standard deviation 8.8 years), indicating that this was a relatively young group of clinicians. The youngest respondent was 23 years old and the oldest respondent was 61 years old. Of the total participants, 27% were consultants, 40% junior trainee doctors were below the consultant grade and 33% were nursing staff and thus the sample was well representative of the clinical oncology workforce in the hospital. The clinical specialties that were represented included all sub-specialties of surgical oncology, medical oncology, radiation oncology, clinical oncology, other allied clinical specialties and specialist oncology nursing. We did not include laboratory-based specialties in this study as they are not directly involved in assessment of PS using the ECOG rating scale. On average, the respondents had spent 4.4 ± 4.2 years in the field of oncology although a minority (14/74, 18.9%) had spent 1 year or less in the field. Table 1 shows the characteristics of the study participants. A small number of respondents (14/74, 16.7%) had international work experience in a country other than India, such as the United Kingdom, USA, Canada, New Zealand, Japan and Australia.

### Overall agreement between raters

**Figure 1.**
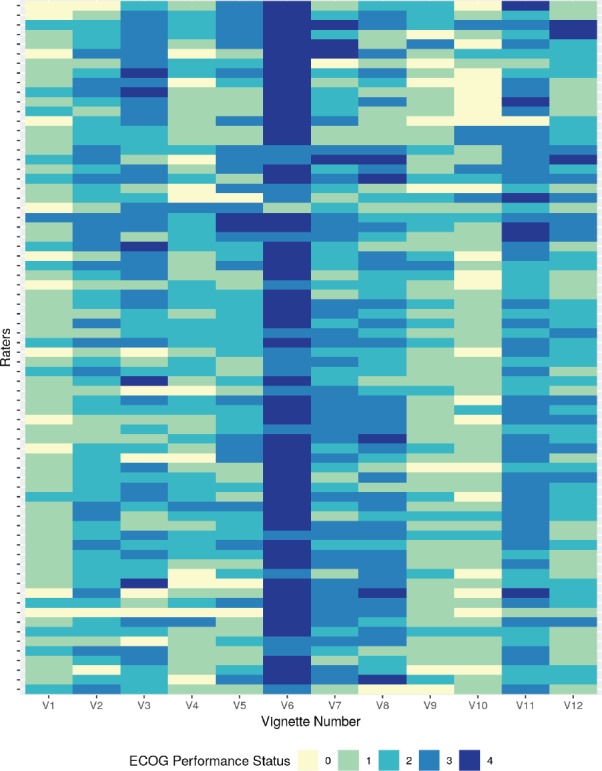
Heatmap of rating given by 72 raters for PS rating of the 12 clinical vignettes (V1–V12).

Overall inter-rater agreement among 72 raters as measured using the Fleiss’s kappa was 0.167 with 95% confidence interval (CI) of 0.042–0.292. The Gwet’s AC1 value for the entire cohort was 0.226 with a 95% CI of 0.162–0.290. The model-based Fleiss kappa calculated using the function provided by Nelson *et al* [13, 15] was 0.156, 95% CI of 0.073–0.238. The overall agreement (or lack thereof) is also graphically depicted in Figure 1 which also shows the major trend of rating among the respondents. The 12 vignettes are represented by V1, V2, V3, V4, …….V12 in Figure 1. The 72 raters are represented by each row. The lesser the ECOG rating or better PS are represented by lighter shades and poorer PS are progressively represented by a darker shade end with deep blue representing ECOG 4. A quick visual inspection will give to the reader an idea about the lack of consensus amongst the raters who participated in the study. As shown in Figure 1, the colour map shows both the heterogeneity in the inter-rater agreement, as well as the kind of rating given to the different vignettes by the same rater. A greater uniformity of the colour as shown for vignette number 6 indicates that similar ratings were given by a greater number of respondents.

### Factors influencing the final ECOG rating

**Figure 2.**
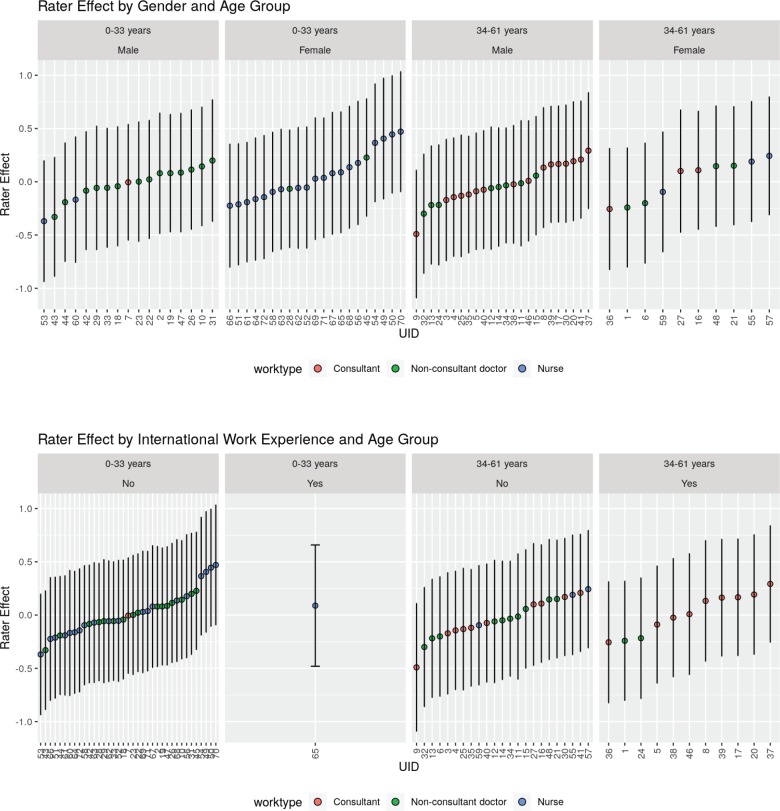

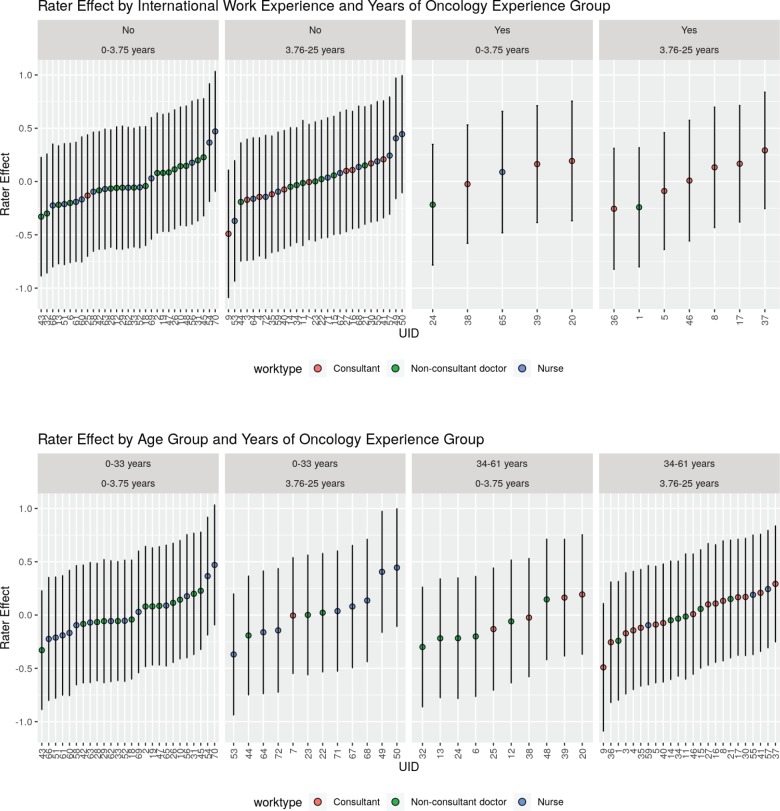
Influence of rater characteristics on the ECOG rating. Bars on the dot represent the 95% CIs. Higher rater-effect indicates a higher rating; however, the differences are not statistically significant given the degree of overlap of the 95% CIs from the dots.

Overall, there was no statistically significant impact of any of the rater characteristics on the rating provided (Table 2 and Figure 2) when a conservative *p* value of 0.004 was considered as a threshold to account for multiple testing. However, nurses tended to rate the PS in vignette 10 worse than the physicians (*p* = 0.009).

There was no significant difference in the agreement in raters when rater characteristics were considered. Table 2 depicts the model-based kappa for agreement, as well as associations for the different subgroups of raters. The kappa values of agreement, as well as association, have overlapping 95% CIs for all rater characteristics, indicating that there is a difference in agreement between raters. The only exception was for clinicians not having ‘international work experience’ in which case the model-based kappa of agreement, as well as that of the kappa of association, was less as compared to those with international work experience. This indicates that there is likely to be an agreement in the rating provided by the respondents with international work experience.

We also investigated if the rating was influenced by the characteristics of the patient in the particular vignette. In order to check this, a CLMM was fitted investigating the effects of three variables; age (geriatric or non-geriatric), gender (male or female) and social desirability (desirable or neutral) of the patient described in the vignette. The final fitted model showed that raters tended to rate the PS worse if the vignette included a patient who was female and lacked in ‘social desirability’ as defined *a priori*.

**Figure 3.**
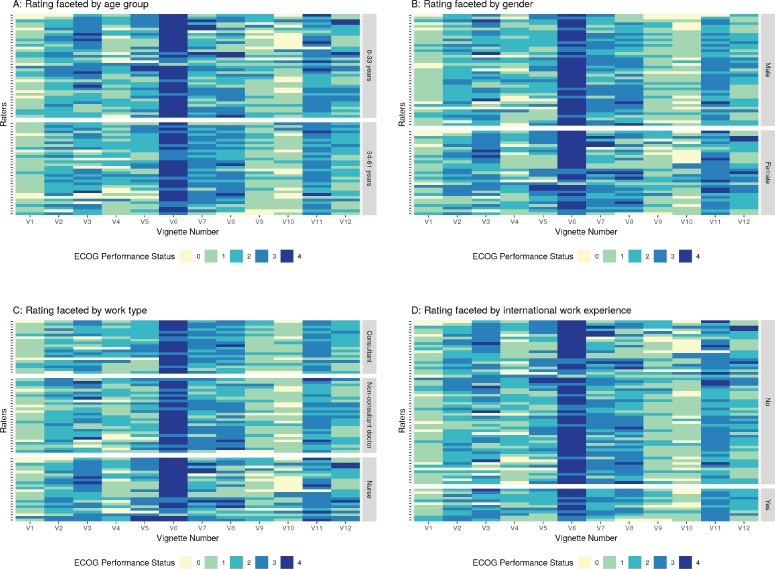

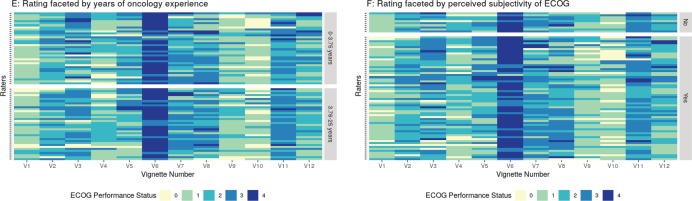
Figure showing the rating given by different raters faceted by the rater characteristics. Panel A: Faceting by age group, Panel B: Faceting as per gender, Panel C: Faceting by work type, Panel D: Faceting by international work experience, Panel E: Faceting by years of oncology experience, Panel F: Faceting by perceived subjectivity in ECOG rating.

Figure 3 represents the agreement between raters when faced by various rater related variables, namely, (a) younger versus older age of the clinician (categorised based on the median age of 33 years), (b) gender of the clinician (men and women), (c) Work type (consultant doctor, non-consultant grade doctor and nursing staff), (d) Presence of international work experience (no versus yes), (e) Longer versus shorter experience of working in the field of oncology (0–3.75 years versus 3.76–61 years, the median number of years of experience in the field of experience being 3.75 and (f) Perceived belief of ECOG rating scale being subjective or not. The 12 vignettes are represented by V1, V2, V3, V4, …….V12 in Figure 3. The 72 raters are represented by each row. The lower the ECOG rating or better PS is represented by lighter shades and poorer PS are progressively represented by a darker shade end with deep blue colour that representing ECOG 4. As is evident from visual inspection of the heatmap, there is considerable variability in ECOG rating and no clear pattern emerges based on the above rater related factors.

There was also significant rater effect and rater number 9 who tended to rate the PS as best was compared to rater number 70 who rated the PS as worse. A graphical exploration of the patterns of rater effect on the rating given revealed that raters who were younger nurses with lesser experience in the field of oncology tended to give a better PS score. Also, younger and less experienced non-consultant clinicians tended to give a poorer PS score.

### Concordance with gold standard rating

Overall concordance with the gold standard rating ranged between 19.4% and 56.9% for the questions with the highest concordance noted for questions 9, 10 and 11 where concordance more than 50% was noted (Table 4). However, in none of the questions did the concordance exceed 60%. While numerical differences were noted between groups, none of the differences were statistically significant at a *p* value of <0.004 (adjusted to account for multiple testing). A graphical description of the differences in the in-group concordance rates is shown in Figure 3 below. The highest concordance was obtained for question 11 for the raters who believed that there was no subjectivity in the ECOG rating. The lowest concordance was obtained for question 5, in the case of nurses. Concordance with the golden rating was generally similar between the consultant and the trainee physicians, except for question 11 for which a larger proportion of consultants were correct. A numerically larger proportion of trainee physicians had concordance with golden rating for Question 1 and 12. Participants with international work experience tended to have higher concordance with the golden rating (except question 9 and 12), though the concordance exceeded 60% in question 11 only.

Chi-square test calculated for proportion of raters with concordance with gold standard rating (*p* values of <0.004 are taken as significant after Bonferroni correction to account for multiple testing)

## Qualitative results

### Importance of rating performance status

Almost all clinicians across various sub-specialties of oncology said that assessing PS was important in cancer care. However, some of the clinicians used it more often than others and they arrived at the final ECOG scores differently:

‘Rating on the ECOG scale is very simple. It is a global score and used all over the world. I use it routinely in my clinical practice. Even for the ‘studies’ that we are conducting and the interventional clinical trials we are part of, we record ECOG PS’ (Consultant Oncologist).

‘I am a surgeon. Anyone who is ECOG 2 or below is unlikely to be a candidate for surgery unless they’re able to improve their PS. If somebody is ECOG 0 or 1, then there’s really not much to worry about in terms of their fitness for surgery. If someone was ECOG 3 or 4, they’re very unlikely to become ECOG 1 or 2. It’s the ones with ECOG 2 who with a bit of nutrition and physiotherapy may be able to be pushed up to ECOG 1. So, if there was someone who was clearly unfit for surgical options then that would be explained to him, saying that although surgery may be technically possible, their PS poses too high a risk. So, then they would be offered the next best treatment option which may be palliative chemotherapy or radiotherapy or just straight up palliative care’ (Consultant Surgical Oncologist).

‘Sometimes we are so short of time, so it’s not possible to ask more questions to conclude about the ECOG PS. If I find it like a borderline 1, 2 then I take it as 2. I take the higher value to be on the safe side’ (Trainee in Radiation Oncology).

Not all clinicians decided on the patient’s PS based on a formal ECOG score, and one doctor expressed that he relies on subjective judgement to arrive at a decision at the start of the clinical encounter:

‘When a patient is entering my room, I can definitely judge whether the patient is fit enough or not’ (Consultant Oncologist).

The value of periodic re-assessment of PS was highlighted by a trainee oncologist.

‘Suppose I feel that on the day I see the patient that he is not doing very well, I may offer him no treatment and refer him to our palliative team. Similarly, if I see that the PS has improved, I may be able to start on some aggressive form of treatment. So, I feel that periodic reassessment of ECOG status is important’ (Trainee in Radiation Oncology).

Disagreements in ECOG score were attributed to lack of training or being less mindful of the general physical characteristics of a patient:

‘Most disagreements (of ECOG score) between raters are with junior colleagues, who have just started in the field of oncology and are still getting used to the ECOG score. This is particularly true for juniors from specialties who do not have basic training in clinical oncology before they come and join as fellows in an oncology centre’ (Consultant Oncologist).

‘Occasionally, the residents do not describe the physical characteristics (of patients). They’ll probably write a ‘PS 1’ because the patient has only a few of the cancer-related symptoms. But the fact that the patient is wheelchair bound or needs a one or two-man support while he comes into the room is ignored’ (Consultant Oncologist).

Some clinicians mentioned the advantages of using other PS rating over ECOG in specific areas of oncology:

‘For children with brain tumours, we use other performance scores like Lansky, etc.’ (Consultant Oncologist)

‘For gynecological cancers, I mostly use ECOG. In Neuro-oncology, we align constantly to Karnofsky because a lot of high-quality evidence has used Karnofsky. So, in our brain database, we have all patients with both ECOG & Karnofsky reported’ (Consultant Oncologist).

However, most oncologists preferred the ECOG performance scale:

‘ECOG seems to be a little easier considering that it is a “0–4 score” as compared to tedious percentages expressed in some of the other assessments’ (Consultant Medical Oncologist).

‘ECOG is very straightforward. The cut-offs for the scale are clear. If I was discussing a patient with another clinician and the patient wasn’t there, and I said the patient was ECOG 2, I think my clinical colleague would understand that’ (Consultant Surgical Oncologist).

## Factors other than performance status that influence variability in decision-making

### Preferences expressed by the patient and family

Several clinicians highlighted that they are also influenced by the preferences expressed by the patient and his or her family members while deciding on treatment options. One surgical oncologist went on to elaborate that

‘I discuss whatever treatment options are available for the particular disease. While discussing with the patient, we describe the pros and the cons associated with each treatment option. Final treatment decisions are made jointly taking the views of the patient and her family’ (Consultant Surgical Oncologist).

### Age of the patient

Treatment decisions may be affected by the age of the patient.

‘Treatment-related decisions for a 35-year-old man who has two small kids will be different from a 75-year-old person who has lived his whole life. He (the older man) may be ready to go but the young person may want to invest that amount of (gestures by hand to show a moderate amount) money to get the 6 months of extra life because for him that may be a lot of time. As a result, many young people take loans for medical treatment, mortgage their property, sell off their sources of income like land/property/cattle/gold and even jeopardise their livelihood for the sake of treatment. At the end of it, even if these are not curative treatments, we cannot play all the roles of the pleader, jury and the judge. We can only give the treatment options, tell the outcomes, what to expect, the cost and then we let them decide’ (Consultant Medical Oncologist).

One of the senior nursing staff stated that for elderly patients where the patient and family had different points of view, he may prefer to respect the way the patient felt:

‘When a 80-year-old patient is undergoing cancer treatment and the quality of life is getting hampered because of the side effects of chemotherapy, certain other things, because of their age or just because of their other co-morbidities, we prefer to go by the decision of the patient though the relatives may want more treatment’ (Nurse in charge).

### Perceived emotional endurance of patient influencing decision-making

One surgical consultant expressed that patient’s psychological make-up also influences treatment decisions.

‘Another thing of course not covered by the ECOG score is what is the patient’s mental makeup. Is he someone who has got the mental strength to go through a rigorous surgery?’ (Consultant Surgical Oncologist).

### Role of past treatment and assessment influencing treatment decisions

‘Sometimes the second opinion that they have obtained already influences the decision. Once everything is available and you know that the diagnosis is in the correct order, you can see things more clearly. I narrow down on the investigations I need, I look at all the other reports and then decide what is required’ (Consultant Medical Oncologist).

### Influence of socio-economic factors on treatment-related discussion

Clinicians are aware of the cost of treatments and the discussions are often personalised based on what the patient can afford as elaborated by a doctor

‘If the patient is coming from the poor socio-economic strata, there is not much point to discuss about 10 or 15 treatment options. Just give them the feasible and possible two options that they can afford. So, “this” is the diagnosis and depending on your current status, “these two or three treatment options are available” and you may choose whichever you want’ (Consultant Medical Oncologist).

### Role of international guidelines influencing treatment protocols and decisions

Solely depending on standardised treatment guidelines to help make decisions was challenged by some of the senior consultants. While discussing international guidelines translating into regional practices, one consultant commented:

‘We keep doing audits and keep looking at patient outcomes every 2 years. This way we get a fairly good idea if the toxicity was more for our patients and if the responses were less (than that published elsewhere). We also look at whether we were able to give treatments on time. By and large, I would say we follow (international guidelines) and there are not too many differences. But we don’t go for the too toxic treatments because we know that we don’t have the logistics and resources to admit all patients when they come with toxicities. So, we abbreviate the treatments to suit our patients and, so naturally, for a disease that has about 85% long term cure rate in western population, in our national centres, we achieve cure rates of about 60%–65%. We still have that much (gestures with both hands) improvements to make, but the fact is if we give very intensive therapy, we will lose 30% of the patient in induction itself. So, we come down on the intensity. That is a judgment we have to make ourselves and proceed. I would say we do modify the protocols, but we do take them from international guidelines that are available’ (Consultant Medical Oncologist).

Another consultant commented about the importance of personalised medicine.

‘I mean at the end of the day it is all about personalised medicine. So, you have to use the guidelines to help you make the best choice for your particular patient. All international guidelines aren’t particularly applicable to our patients in our context. I look more to guidelines that are developed in this country. So yes, guidelines are important, they give you the general framework of how you treat. But, you actually do what is going to be best, individualised to every patient’ (Consultant Surgical Oncologist).

One of the consultants highlighted that she has been involved in developing national guidelines:

‘We had developed the Indian Council of Medical Research (ICMR) guidelines for which I have been part of (the team) and now we have something called the National Cancer Grid for which I have written guidelines as well. Now we are trying to make the guidelines a little more broad-based. I do not know if people will follow them or not, but the intention is that we should have something’ (Consultant Medical Oncologist).

### Juxtaposition of quantitative and qualitative results

**Figure 5.**
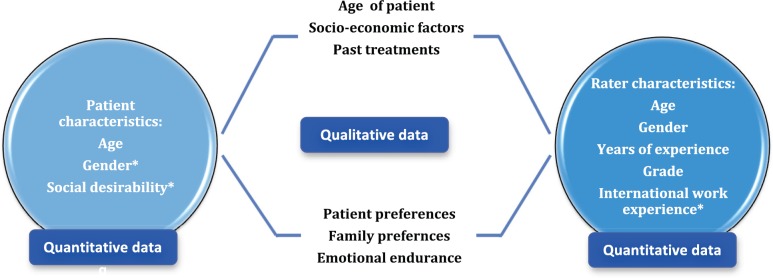
Juxtaposition of quantitative and qualitative findings. *Statistically significant at *p* < 0.004.

The quantitative data looked into rater characteristics, such as age of the clinician, gender of the clinician, number of years of experience in oncology, grade of work and international work experience as potential factors that could have had a bearing on the rating pattern and found that only international work experience was shown to have improved agreement between raters. The patient characteristics were studied as well. Being a lady patient in the vignette was associated with poorer rating of PS. On the other hand, being perceived as someone with socially desirable characteristics made them have a better PS score. The qualitative in-depth interviews showed that non-PS factors made a significant impact on treatment-related discussions as well. The clinicians highlighted that ‘age of the patient’, ‘perceived socio-economic status of the patient’, ‘emotional endurance of the patient’ and ‘preferences expressed by the patient and the family’ all influenced decision-making in oncology. In Figure 5, we have juxtaposed the findings from the qualitative arm of our study with the quantitative results.

## Discussions

The current study showed that there is poor agreement between raters for a majority of case vignettes on a wide range of oncological conditions. The agreement was slightly better with improved training and more experienced clinicians, although it never crossed 60%. Variability in ECOG rating has been reported by Sorensen *et al* [5] and Ando *et al* [18]. In studies using less number of raters, the consistency of ECOG rating was better [6]. Patients who were perceived to have socially desirable qualities were deemed to have better PS. In low- and middle-income country (LMIC) settings, where resources can be limited, oncology clinicians often have to make important decisions quickly using yet unexplored heuristic patterns. ‘Heuristics’ are cognitive shortcuts that are used in situations of high complexity or uncertainty [19] or when the time for individual decision-making is short [20], both of which are common factors in a busy oncology clinic or multidisciplinary team meetings (MDT). There has been some work on how decisions are processed by individuals. Jumping to conclusions can be efficient if conclusions are likely to be correct and the cost of an occasional mistake is acceptable, especially when the ‘jump’ saves much time and effort. However, jumping to conclusions is unacceptable when the stakes are high and there is less time to collect more information. In the second scenario, intuitive errors are probable [21]. The causes of inter-observer variations could also be linked to an affective heuristics error. A doctor may allow positive feelings towards a patient to influence his clinical judgment because he wishes the patient well and a symptom may be interpreted benignly when a more ominous interpretation is valid [22]. In-depth qualitative interviews of clinicians elicited several non-disease related factors as socio-economic background, past treatment-related factors, patient preferences and emotional factors that often influenced treatment-related discussions. Contextual factors have been shown to impact on decision-making in other field of medicines [23–25]. Qualitative data presented in the study suggest that clinicians reported that treatments were perceived to be more beneficial by younger patients even when associated with higher probability of social, medical and financial adverse events. Similar studies done on physicians who answered a questionnaire about treatment decisions on elderly cardiac patients with multiple comorbidities, found that physician while treating this group of elderly patients depended more on their own personal experience and patient preferences than on standardised guidelines [26]. Prospect theory predicts that in situations of perceived loss, more riskier decisions may be made [27] and younger patients often do so more than the elderly [28]. Social factors do influence the decision-making process [29]. The nature of professional training may impact decision-making and in fact, there is some data to suggest that for non-specific chest pain, cardiologists and internists may differ in terms of investigations requested [29]. It would be interesting to study similar patterns of decision-making in different professional groups in oncology in future. There have been some previous reports on concordance rates between raters from various specialties of medicine and being blind to the initial report of a patient can reduce the concordance rate of the final report [23, 30]. There have even been suggestions of blinding clinicians to contextual factors as is often done in forensics to avoid cognitive bias [31]. There needs to be more research on cognitive bias in decision-making [32]. In the current study, the oncology clinicians also considered the age of the patient while deciding on treatment options. Our finding that oncologists reporting PS of female patients less favourably than male patients has been reported previously [33].

The perception of the respondents in the current study that patients often get led by the choice of the clinicians has been found in other settings but it was noted that non-collaborative decision-making was associated with increased non-adherence to treatment in the longer run [34]. One Australian study observed that patients tend to report their PS better than the oncologists and there was good agreement between raters for both the PS scales [35]. Another study prospectively looked into the inter-observer variation in recording the ECOG score or Karnofsky Performance (KP) score between two oncologists assessing 209 consecutive patients and concluded high degree of correlation [*k* = 0.914], [36]. The respondents of our study mentioned that clinicians would like to offer their patients a choice regarding the treatment options available. Given that at the onset of treatment, patients are often slightly over optimistic about their own outcome, this should be kept in mind while discussing treatment choices. Other studies have also looked into the use of patient scored PS. One study reported 51% agreement in the PS scores between clinicians and patients [33]. The same study also suggested oncologist-scored PS scores were marginally more predictive of survival. The better correlation of physician-scored PS and the survival outcome was supported in another study by Ando *et al* [18]. One group highlighted the importance of using patient-rated PS in advanced malignancies [37].

The strengths of the current study include recruiting a range of clinicians reflecting a real-life oncology hospital and following a mixed method of enquiry. The qualitative data was collected and analysed following the framework of the COREQ guidelines [12]. The data were collected by researchers who were not part of the hospital. Clinicians were interviewed in private so that they could open up freely. One limitation of the study was that it was conducted in a single large oncology centre and the findings may not be generalisable outside the setting. Another important limitation of the study is that it used clinical vignettes over real-life actors or real patients. Although vignettes were designed in such a way that they represent real-life patients and were close approximations to actual case scenarios, it is important to acknowledge that evaluating a ‘vignette’ is not the same as health professionals evaluating a real patient. As vignettes do not come with the conscious and unconscious emotions of having to deal with a human being, some of the decisions made while deciding about vignettes may be more extreme than real-life situations. Previous research that compared trained actors as a proxy to the clinical situation, clinical case vignettes and chart review summaries, showed that case vignettes were a close second [7] after real-life actors but were better than case review summaries. The authors acknowledge that vignettes represent reality only to a certain extent.

In resource-poor settings, treatment guidelines are often adapted to the individual patient and his or her circumstances. If this diversity is acknowledged formally, treatment-related decisions will less often be left to a matter of ‘chance’. Better training and more experience in the field of oncology may lead to more agreement on the ECOG PS, better treatment-related decisions and more homogenous care delivery. Clinicians will certainly benefit from training on how to adapt treatment options to specific individuals. There is literature to suggest that educating clinicians on probabilistic reasoning will achieve more consistency in assessing a clinical situation [38]. Future research should be conducted on how to train doctors to think about solving complex problems and make them aware of potential sources of unconscious biases in thinking.

## Conclusion

ECOG PS scoring is a widely used instrument for assessing PS in oncology. However, there is considerable variability in the way people rate patients. Further training and education, particularly of junior clinicians on the use of ECOG may improve their agreement on the ECOG sore. Decision-making in oncology is often influenced by the clinician’s perception of the patient and his or her circumstances over and above the PS. Training in oncology should place emphasis on looking at the person beyond the tumour and help clinicians deliver reasonable cancer care that is feasible and acceptable to the patient.

## Conflicts of interest

The authors do not have any conflicts of interest to declare.

## Funding and support

The authors thank the Newcastle Business School, University of Newcastle for support the visiting interns (Chantalle van Zanten and Joe Parry) through the Global Experience Opportunity programme to work with the research team in the Tata Medical Centre, Kolkata. The other authors were supported by their employer, Tata Medical Centre, Kolkata, for conducting the research.

## Figures and Tables

**Figure 4.**
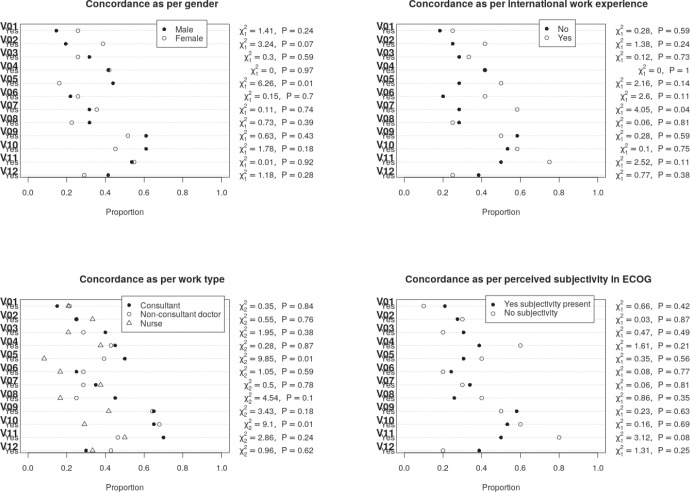
Showing the concordance of the different groups for the different questions. Proportion in which the rating was concordant with the golden rating shown in the dot plots.

**Table 1.** Demographic characteristics of study participants.

Variable	Parameter	Value (*n* = 74)
Age (years)	Mean ± standard deviation (range)	33.4 ± 8.8 (23–61)
Gender	Female (%)	31 (43.1%)
Year of oncology experience	Mean (standard deviation)	4.4 ± 4.2 (1–25)
Work type	Consultant physician (%)	20 (27.8%)
	Trainee physician (%)	28 (38.9%)
	Nurses (%)	24 (33.3%)
International work experience	Yes (%)	14 (16.7%)
Perceived subjectivity of ECOG (respondents were asked if in their opinion ECOG PS rating was subjective)	No subjectivity (%)	10 (13.9%)

**Table 2.** Table showing the model-based kappa of agreement and kappa of association for the rating provided by different groups of raters. The figures in parentheses represent the 95% CI of the estimate.

Variable	Parameter	Kappa of Agreement	Kappa of Association
Age group	0–33 years	0.15 (0.07–0.23)	0.33 (0.18–0.48)
	>33 years	0.18 (0.09–0.27)	0.38 (0.23–0.53)
Gender	Male	0.19 (0.10–0.29)	0.40 (0.25–0.56)
	Female	0.12 (0.05–0.19)	0.28 (0.14–0.42)
Years of oncology experience	0–3.75 years	0.14 (0.06–0.21)	0.31 (0.16–0.45)
	>3.75 years	0.18 (0.09–0.26)	0.38 (0.22–0.53)
Work type	Consultant	0.21 (0.11–0.30)	0.43 (0.27–0.58)
	Non-consultant physician	0.18 (0.09–0.26)	0.37 (0.22–0.53)
	Nurse	0.12 (0.05–0.20)	0.28 (0.14–0.42)
International work experience	Yes	0.02 (0.00–0.04)	0.05 (0.01–0.10)
	No	0.15 (0.07–0.24)	0.34 (0.19–0.49)
Interpretation of ECOG	Subjectivity present	0.15 (0.07–0.24)	0.34 (0.19–0.49)
	No subjectivity	0.16 (0.07–0.24)	0.34 (0.19–0.49)

**Table 3.** Influence of the patient characteristics on the PS rating. The model incorporated rater as a random variable.

Variable	Parameter	Coefficient	Standard Error	p value
Case Age	Non-geriatric	0.06	0.18	0.73
Case Gender	Male	0.56	0.22	<0.01
Case Social Desirability	Neutral	−0.0142	0.17	0.93
	Desirable	−1.39	0.17	<0.01

## Appendix I

**Table:** ECOG Performance Status*

Grade	ECOG
0	Fully active, able to carry on all pre-disease performance without restriction
1	Restricted in physically strenuous activity but ambulatory and able to carry out work of a light or sedentary nature, e.g. light housework, office work
2	Ambulatory and capable of all selfcare but unable to carry out any work activities. Up and about more than 50% of waking hours
3	Capable of only limited selfcare, confined to bed or chair more than 50% of waking hours
4	Completely disabled. Cannot carry on any selfcare. Totally confined to bed or chair
5	Dead

* As published in *Am J Clin Oncol*: Oken MM, Creech RH, and Tormey DC* et al. (1982)* **Toxicity and response criteria of the eastern cooperative oncology group** *Am J Clin Oncol* **5*** 649–655.*

## Appendix II

The case vignettes, [**reasoning behind each vignette**], (gold standard PS and ‘social desirability factors’ of the patient as decided by the researchers who designed the vignettes):

1. An 84-year-old patient of non-small cell lung cancer (NSCLC) who is self-caring but usually spends most of his time watching TV and reading newspaper. He enjoys walking in the local park in the afternoon with his grand-daughter but finds it difficult to be as active as he was 10 years ago. What is the ECOG rating of this patient?
  **[*This question tests the assumption whether this sedentary level of activity should be considered slightly restricted for an 84-yearold patient, hence he is considered PS-1 or being significantly restricted in more than 50% of the time, hence he is considered PS-2.*]**
  (Gold standard rating PS-2; Rated as ‘socially desirable’ patient.)
2. A 35-year-old salesman and amateur footballer is diagnosed with glioblastoma multiforme (GBM), suffering expressive dysphasia and loss of balance, is able to get up in the morning and get dressed. In the past 2 months, he has stopped working. He watches television for most of the day and manages to feed himself. In the evening, he goes out with his wife for a short walk for 15–20 minutes. What is the ECOG status of the person?
  **[*This question was designed to study the clinician’s way of thinking. It tries to explore if the premorbid PS or current PS influences the clinician in deciding on the treatment options.8*]**
  (Gold standard rating PS-3; Rated as ‘socially desirable’ patient.)
3. A 55-year-old lady with osteoarthritis who was on a wheelchair to help her mobilise inside the hospital came into the clinic. She uses a crutch when she is in the shop she runs in the local mall. She enjoys knitting and playing with her grandson during weekends. She is diagnosed with advanced lung cancer. How would you rate the ECOG performance status of the patient?
  **[*This question intends to find out how clinicians would interpret the inability to continue with active lifestyle due to a totally unrelated benign condition.*]**
  (Gold standard rating PS-2; Rated as ‘socially neutral’ patient.)
4. A 77-year-old retired head teacher spends a lot of time usually reading newspapers and books. Sob on walking 50–100 yards and been like this for years because of chronic obstructive pulmonary disease (COPD). He gets dressed in the morning, and goes downstairs, watches television or reads books. In the evening he has dinner with his wife. He rests about an hour in the afternoon and goes to the nearby bus stop to chat with some of his friends on couple of evenings every week. Occasionally, he goes out for shopping to the supermarket in his car which is driven by his driver. He is now diagnosed with bladder cancer. What is the ECOG rating of the patient?
  **[*The question tests if the rater allow the age-related normal decrease inactivity levels into consideration when interpreting PS.*]**
  (Gold standard rating PS-2; Rated as ‘socially neutral’ patient.)
5. A 58-year-old male patient, with BMI of 22, who lives alone has been diagnosed with metastatic non-small cell lung cancer. His main problems are cough interfering with his sleep and weight loss of 12 kilograms. He has background COPD and his exercise tolerance is limited to 250 yards on the flat. He rests in bed for 2 hours every afternoon. He has been referred for consideration of palliative treatment. How would you rate his ECOG PS?
  **[*This question is trying to find out whether rapidly progressive health deterioration in patients affect the way clinicians think and interpret PS. This patient is rapidly progressing towards PS-3.*]**
  (Gold standard rating PS-2; Rated as ‘socially neutral’ patient.)
6. A 66-year-old lady, previously fit, and who used to work as an officer in a bank, has developed rapidly progressive hemiparesis over the last 3 days, causing her to be bed-bound. Her cognition, comprehension and speech are intact. CT shows a contrast enhancing right parietal tumour. How would you rate her ECOG PS?
  **[*This case examines whether clinicians would disregard the acute deterioration of PS due to the underlying disease. This will affect the management this patent is going to have.*]**
  (Gold standard rating PS-3; Rated as ‘socially desirable’ patient.)
7. A 55-year-old lady is due her 6th cycle of 2nd line palliative docetaxel chemotherapy for metastatic breast cancer. She was very lethargic for the first 10 days of the last cycle and slept for most of the day during this period. However, last week she started to feel better, but still quite weak and resting in between mild household work. Her husband is doing most of the housework. (There has been some response in the post cycle 3 scan). How would you rate the patient’s ECOG PS?
  **[*This is a common scenario in an Oncology clinic. Do we take the PS when it is at its worst and stop the next cycle of chemo or proceed to #6 as planned as there is some response in the scan.*]**
  (Gold standard rating PS-2; Rated as ‘socially desirable’ patient.)
8. A 58-year-old lady who works as a teacher has ascites and pedal oedema. She feels tired all the time, has lost her appetite and has lost 10 kg of her body weight. Her daughters live nearby and help her with housework. She has been diagnosed with ovarian cancer and is not deemed suitable or fit for primary surgery. She has been referred for consideration of chemotherapy. How would you rate the patient’s ECOG PS?
  **[*This question examines the heuristics of clinical decision-making. Do we call her PS-3 and offer palliative care alone or do we call this PS-2 and offer the patient chemotherapy as ovarian cancer can be fairly chemo-sensitive?*]**
  (Gold standard rating 2; Rated as ‘socially neutral’ patient.)
9. A 38-year-old lady was treated with concomitant chemo-radiotherapy followed by brachytherapy for cervical cancer 2 years ago. Since then, she has suffered from chronic diarrhoea and unpredictable bowels. She would never want to be far from the toilet and therefore almost never goes out of her house. She is now being discussed at the MDT for management of recurrent cervical tumour. She can do all her housework. What is her ECOG PS?
  **[*This vignette is trying to identify how clinicians are going to interpret the self-confinement of the patient.*]**
  (Gold standard rating PS-1; Rated as ‘socially neutral’ patient.)
10. A 71-year-old politician, who was also a good orator and the local MP, had T4 laryngeal cancer that was treated with laryngectomy followed by post-operative radiotherapy 2 years ago. He has since retired and spends his time in the study and his garden. He is now diagnosed with T2 tumour of the posterior tongue and being discussed at your MDT. How would you rate the ECOG PS of the patient?
  **[*This question examines the effect of ‘difficult to treat’ relapsed disease in interpreting PS.*]**
  (Gold standard rating PS-1; Rated as ‘socially neutral’ patient.)
11. A 67-years-old gardener attends clinic with his wife and son who is 37 years old. He is currently diagnosed with anaplastic astrocytoma of the brain and is suffering severe expressive dysphasia and has significant lack of balance. He is housebound and watches television all day. He goes for a short walk down the street holding onto his son every day. What is his ECOG PS? Is he suitable for an intervention trial with ECOG PS cut-off of 2?
  **[*In this question, we are trying to see if access to particular trial drugs or treatment thought to be beneficial other than the standard treatment options (available off trial) distorts the way we assign ECOG PS?*]**
  (Gold standard rating PS-3; Rated as ‘socially neutral’ patient.)
12. A 67-year-old lung cancer patient presents with a local relapse after previous radical radiotherapy to his lung 2 years ago. He restricts himself within the premises of his own house due to breathlessness on exertion. He can drive a car if needed to the local shop to pick up his shopping. He can’t have chemotherapy due to poor renal functions and is not suitable for targeted therapies. A new re-irradiation of lung trial is recruiting patents of locally relapsed previously treated lung cancers if the patients have a PS 0 or 1. Does he qualify for the trial or should he be managed with best supportive care alone? How would you rate the patient’s ECOG PS?
  **[*This question intends to see if availability of trials influence clinical decision-making.*]**
  (Gold standard rating PS-1; Rated as ‘socially neutral’ patient.)

## Appendix III

**Table 4.** Percentage of raters giving a specific rating for each question as per the rater characteristics. Q: Question number. PS: ECOG PS score, Age group: Age split by median age, Experience group: Years of oncological experience by median, M: Male, F: Female, C: Consultant, T: Trainee Physicians, N: Nurses, Y: Yes, N: No. Shaded table rows represent the golden rating.

Q	PS	Age Group		Gender		Work Type			International work experience		Years of oncology experience	
		0-33 (38)	> 33 (34)	M (41)	F (31)	C (20)	T (28)	N (24)	Y (12)	N (60)	0–3.75 (36)	>3.75 (36)
1	0	21.1%	11.8%	17.1%	16.1%	15.0%	14.3%	20.8%	16.7%	16.7%	13.9%	19.4%
	1	52.6%	73.5%	68.3%	54.8%	70.0%	64.3%	54.2%	58.3%	63.3%	63.9%	61.1%
	2	23.7%	14.7%	14.6%	25.8%	15.0%	21.4%	20.8%	25.0%	18.3%	22.2%	16.7%
	3	2.6%	0.0%	0.0%	3.2%	0.0%	0.0%	4.2	0.0%	1.7%	0.0%	2.8%
2	0	2.6%	5.9%	4.9%	3.2%	10.0%	0.0%	4.2%	0.0%	5.0%	0.0%	8.3%
	1	18.4%	20.6%	14.6%	25.8%	25.0%	17.9%	16.7%	25.0%	18.3%	25.0%	13.9%
	2	52.6%	44.1%	61.0%	32.3%	40.0%	57.1%	45.8%	33.3%	51.7%	50.0%	47.2%
	3	26.3%	29.4%	19.5%	38.7%	25.0%	25.0%	33.3%	41.7%	25.0%	25.0%	30.6%
3	0	0.0%	17.6%	9.8%	6.5%	15.0%	10.7%	0.0%	8.3%	8.3%	8.3%	8.3%
	1	18.4%	8.8%	17.1%	9.7%	10.0%	21.4%	8.3%	0.0%	16.7%	19.4%	8.3%
	2	21.1%	38.2%	31.7%	25.8%	40.0%	28.6%	20.8%	33.3%	28.3%	25.0%	33.3%
	3	50.0%	32.4%	34.1%	51.6%	35.0%	28.6%	62.5%	50.0%	40.0%	33.3%	50.0%
	4	10.5%	2.9%	7.3%	6.5%	0.0%	10.7%	8.3%	8.3%	6.7%	13.9%	0.0%
4	0	15.8%	14.7%	17.1%	12.9%	10.0%	14.3%	20.8%	0.0%	18.3%	19.4%	11.0%
	1	39.5%	38.2%	36.6%	41.9%	35.0%	42.9%	37.5%	41.7%	38.3%	36.1%	41.7%
	2	42.1%	41.2%	41.5%	41.9%	45.0%	42.9%	37.5%	41.7%	41.7%	38.9%	44.4%
	3	2.6%	5.9%	4.9%	3.2%	10.0%	0.0%	4.2%	16.7%	1.7%	5.6%	2.8%
5	0	2.6%	5.9%	4.9%	3.2%	5.0%	3.6%	4.2%	0.0%	5.0%	5.6%	2.8%
	1	26.3%	35.3%	31.7%	29.0%	25.0%	35.7%	29.2%	33.3%	30.0%	22.2%	38.9%
	2	26.3%	38.2%	43.9%	16.1%	50.0%	39.3%	8.3%	50.0%	28.3%	33.3%	30.6%
	3	39.5%	20.6%	19.5%	45.2%	20.0%	21.4%	50.0%	16.7%	33.3%	38.9%	22.2%
	4	5.3%	0.0%	0.0%	6.5%	0.0%	0.0%	8.3%	0.0%	3.3%	0.0%	5.6%
6	1	2.6%	0.0%	0.0%	3.2%	0.0%	0.0%	4.2%	0.0%	1.7%	2.8%	0.0%
	3	13.2%	35.3%	22.0%	25.8%	25.0%	28.6%	16.7%	41.7%	20.0%	25.0%	22.2%
	4	84.2%	64.7%	78.0%	71.0%	75.0%	71.4%	79.7%	58.3%	78.3%	72.2%	77.8%
7	0	2.6%	0.0%	0.0%	3.2%	0.0%	0.0%	4.2%	0.0%	1.7%	2.8%	0.0%
	1	21.1%	11.8%	14.6%	19.4%	5.0%	21.4%	20.8%	8.3%	18.3%	13.9%	19.4%
	2	39.5%	26.5%	31.7%	35.5%	35.0%	28.6%	37.5%	58.3%	28.3%	38.9%	27.8%
	3	26.3%	61.8%	53.7%	29.0%	60.0%	50.0%	20.8%	33.3%	45.0%	38.9%	47.2%
	4	10.5%	0.0%	0.0%	12.9%	0.0%	0.0%	16.7%	0.0%	6.7%	5.6%	5.6%
8	0	0.0%	2.9%	0.0%	3.2%	0.0%	3.6%	0.0%	8.3%	0.0%	0.0%	2.8%
	1	31.6%	8.8%	17.1%	25.8%	5.0%	14.3%	41.7%	16.7%	21.7%	27.8%	13.9%
	2	21.1%	35.3%	31.7%	22.6%	45.0%	25.0%	16.7%	25.0%	28.3%	22.2%	33.3%
	3	39.5%	47.1%	46.3%	38.7%	45.0%	50.0%	33.3%	50.0%	41.7%	41.7%	44.4%
	4	7.9%	5.9%	4.9%	9.7%	5.0%	7.1%	8.3%	0.0%	9.3%	8.3%	5.6%
9	0	13.2%	8.8%	9.8%	12.9%	5.0%	7.1%	20.8%	16.7%	10.0%	11.1%	11.1%
	1	50.0%	64.7%	61.0%	51.6%	65.0%	64.3%	41.7%	50.0%	58.3%	61.1%	52.8%
	2	31.6%	26.5%	29.3%	29.0%	30.0%	25.0%	33.3%	33.3%	28.3%	25.0%	33.3%
	3	5.3%	0.0%	0.0%	6.5%	0.0%	3.6%	4.2%	0.0%	3.3%	2.8%	2.8%
10	0	34.2%	23.5%	29.3%	29.0%	20.0%	28.6%	37.5%	33.3%	28.3%	27.8%	30.6%
	1	44.7%	64.7%	61.0%	45.2%	65.0%	67.9%	29.2%	58.3%	53.3%	58.3%	50.0%
	2	13.2%	5.9%	9.8%	9.7%	15.0%	3.6%	12.5%	8.3%	10.0%	5.6%	13.9%
	3	7.9%	5.9%	0.0%	16.1%	0.0%	0.0%	20.8%	0.0%	8.3%	8.3%	5.6%
11	0	2.6%	0.0%	2.4%	0.0%	0.0%	0.0%	4.2%	0.0%	1.7%	2.8%	0.0%
	1	2.6%	2.9%	2.4%	3.2%	5.0%	0.0%	4.2%	0.0%	3.3%	2.8%	2.8%
	2	36.8%	29.4%	39.0%	25.8%	25.0%	46.4%	25.0%	25.0%	35.0%	41.7%	25.0%
	3	47.4%	61.8%	53.7%	54.8%	70.0%	46.4%	50.0%	75.0%	50.0%	44.4%	63.9%
	4	10.5%	5.9%	2.4%	16.1%	0.0%	7.1%	16.7%	0.0%	10.0%	8.3%	8.3%
12	1	36.8%	35.3%	41.5%	29.0%	30.0%	42.9%	33.3%	25.0%	38.3%	33.3%	38.9%
	2	34.2%	52.9%	43.9%	41.9%	60.0%	39.3%	33.3%	58.3%	40.0%	38.9%	47.2%
	3	21.1%	11.8%	14.6%	19.4%	10.0%	17.9%	20.8%	16.7%	16.7%	19.4%	13.9%
	4	7.9%	0.0%	0.0%	9.7%	0.0%	0.0%	12.5%	0.0%	5.0%	8.3%	0.0%
